# Influence of cardiac rehabilitation in Primigravida with spontaneous coronary artery dissection during postpartum

**DOI:** 10.1186/1755-7682-7-20

**Published:** 2014-05-06

**Authors:** Mariana de Carvalho Pinto, Regina Celi Trindade Camargo, José Carlos Silva Camargo Filho, Cristina Elena Prado Teles Fregonesi, Ana Clara Campagnolo Real Gonçalves, Luiz Carlos de Abreu, Luiz Carlos Marques Vanderlei, Roselene Modolo Regueiro Lorençoni

**Affiliations:** 1 Student of Lato-sensu Postgraduation programme in the sector of Cardiovascular Rehabilitation in the Study Centre in Physiotherapy, Rehabilitation of Faculdade de Ciência e Tecnologia, UNESP, Presidente Prudente, Brazil; 2 Department of Health Science, Faculdade de Medicina do ABC, Santo André, SP, Brazil; 3 Department of Physioterapy in the Study Centre in Physiotherapy, Rehabilitation of Faculdade de Ciências e Tecnologia, UNESP, Rua Roberto Simonsen, 300, Centro Educacional, 19060-900 Presidente Prudente, SP, Brazil

**Keywords:** Cardiac rehabilitation, Spontaneous coronary artery dissection, Aerobic exercises

## Abstract

**Background:**

The physical exercise consists of trainable physical abilities such as strength and endurance. It can be inferred that the individual cardiac patient is dependent on it as an associated therapy to the drug treatment for a rapid and lasting improvement of their overall clinical status

**Case presentation:**

The patient – with Spontaneous Coronary Artery Dissection Postpartum period – was subjected to 21 sessions of cardiac rehabilitation. A physical evaluation was performed, before and after the treatment period, for data collection: anthropometric values, flexibility, aerobic capacity and strength of grip.

**Conclusion:**

The patient had a positive response in aerobic capacity, flexibility and grip strength and the anthropometric values were kept in short term rehabilitation.

## Background

When considering that practice of physical exercise promotes positive acute and chronic effects to improve the individual cardiac patient, for example, the decrease in blood pressure (BP). Thus, the relevance of its prescription connected to drug therapy is justified when targeting a faster and lasting improvement of their general medical condition [1].

Moreover, changes in habits and lifestyle are also part of the rehabilitation process, such as salt restriction diet, smoking and alcoholic cessation, together with drug administration and supervised physical rehabilitation [2,3].

Among the known cardiopathies, Spontaneous Coronary Artery Dissection (SCAD) is a rare condition, responsible for 0.1% to 1.1% of the myocardial ischemia and affects, in general, younger patients during the postpartum period and without history of atherosclerotic disease, which accounts for two thirds of the patients [4,5].

Therefore, considering that SCAD is one of the indicated disorders for treatment through cardiovascular rehabilitation and the physiological and functional benefits provided by it, it was found appropriate to analyse the influence of aerobic training on the anthropometric characteristics, flexibility, aerobic capacity and grip strength in a holder of the above disorder.

## Case presentation

The patient checked in the sector of Cardiovascular Rehabilitation in the Study Centre in Physiotherapy and Rehabilitation of *Faculdade de Ciências e Tecnologia – UNESP,* in Presidente Prudente, Brazil, following medical recommendation, in September 2011. She was 36 years old, white, primigravida, diagnosed with SCAD ten days postpartum, neither showing previous cardiopathy nor history of drug use, no problems during prenatal, and she was subjected to a cesarean delivery in April 2010.

Ten days after delivery, the patient had a sudden illness and was sent to Intensive Care Unit, where she suffered an Acute Myocardial Infarction (AMI). A Diagnostic Cardiac Catheterization was performed followed by a Therapeutic Cardiac Catheterization, on which an “STENT” implant was placed in the anterior descending coronary artery.

One month after the AMI the patient was subjected to another Diagnostic Cardiac Catheterization and obstruction of 90% “intra-STENT” was found; the diagonal artery (DA) totally obstructed in the middle third and the circumflex artery (C×A) showing a target lesion of 80% in the middle third.

In November 2010 the patient was subjected to a Cardiac Resonance, and borderline left ventricular function equal to 52% was found due to a hypokinesia of the basal anterior and lateral walls of the left ventricle. An Angiotomography showed the presence of significant single-vessel luminal reduction; there was in C×A the presence of dissection of the distal left main coronary artery and DA proximal; the presence of pervious “STENT” and no luminal reduction in DA were also found.

In December 2010 the patient submitted to coronary artery bypass surgery (CABG) with the placement of a mammary artery bypass and coronary artery bypass.

In January 2011 the Angiotomography was re-done and the venous graft was occluded in its emergency of anterior face of the ascending aorta. In July 2011 an exam stress/rest Myocardial was performed in which a persistent hipoperfusion in the anterolateral wall of the left ventricle was observed, with discreet component of transience associated in the basal area beyond a low degree depressed contractile function of the left ventricle.

A Holter was done, in which dominant sinus rhythm, absence of arrhythmias and heartbeat rate varied from 48 to 110 beats per minute (bpm), with an average of 70 bpm. The Ecocardiogram found mild mitral regurgitation and ejection fraction of 63%.

In the clinical examination, in September 2011, she reported great limitation in performing daily life activities, tiredness, dizziness when lowering and standing up, the right upper limb was weak if compared to the left and she described a discomfort under the right breast triggered by effort, which would get better at rest and worse if the triggering activity would not stop. She took 125 mg of Atenolol, 100 mg of acetylsalicylic acid and 10 mg of atorvastatin per day.

## Materials and methods

### Physiotherapeutic assessment

Before and after the intervention, a physical assessment was performed through the tests mentioned below.

The anthropometric data were collected – through taking the weight in kilograms (Welmy R/I 200 – Brazil) and the height in centimeter (stadiometer: Sanny-Brazil) – so that the Body Mass Index (BMI) could be obtained. The calculation of total body fat percentage was done using the skin fold measures (adipometer: Sanny-Brazil ), according to the POLLOCK protocol (1980) [6], in order to obtain the complementary data to the equation proposed by Siri (1961) [7]. The visceral fat index was obtained through measuring the body circumferences of the waist and hips, using a flexible tape with 0.1 cm precision (Sanny-Brazil), which provided a ratio between them [8].

As a means to assess aerobic capacity, the Six-minute Walk Test was used [9]. Flexibility was assessed in an indirect way through the sit-and-reach test, using Wells’ Bench (Sanny-Brazil) [10]. As an indicator of the total body strength, the participant’s grip strength was obtained with a handgrip dynamometer (CROWN® - Brazil) [11].

The statistical treatment was descriptive, done through simple frequencies and percentages.

### Intervention protocol

Cardiovascular rehabilitation sessions were done, three times a week, lasting for one hour. This came to a total of 21 sessions. These were divided, respectively, measurement of vital signs, warming up, aerobic activity and cooling down. In the first phase of the session cardiac frequency (CF) was assessed through auscultation for 15 seconds which were multiply by four in order to obtain the results in bpm; BP was taken in an indirect way (non-invasive) through brachial artery auscultation with a sphygmomanometer and an adult stethoscope.

Warming up would last for 20 minutes when calisthenic exercises were done in order to maintain and improve coordination and flexibility through stretching followed by exercises that stimulate global and local muscle either in upper limbs or in lower limbs.

Next phase consisted of aerobic activity for 30 minutes, divided in 15 minutes on a treadmill and 15 minutes on an exercise bike, performed randomly in each session. In this phase it is necessary to mention that there are two moments in which hemodynamic is checked, in the fourth and tenth minutes of activity in the cycle ergometers. In the last phase the patient was stimulated to lie down and rest for four minutes so that the hemodynamic would go back to a value close to the initial and the CF was measured at the end of this period.

As a complement to the physiological response, BORG’s scale was applied according to Borg (1982) [12], along with measuring hemodynamic. The exercise intensity was maintained between 45 and 60% of the Heart Rate Reserve (HRR), moderate intensity, according Mezzani et al., (2013) [13] once, based previously described tests, the patient was classified as high risk according to Buchler and cols, (1996) [14].

## Results

At the end of the interventions, the patient underwent another ecocardiography that, besides having shown segmental myocardial damage of the left ventricle, left ventricular diastolic dysfunction level I, mild mitral regurgitation and ejection fraction of the left ventricle equal to 57%, did not bring up any improve in relation to the previous test, according medical report.

In the sixteenth session, a Holter lasting approximately 24 hours was done, which shows: transitory periods of discrete bradycardia during night rest, only one ventricular extrasystole during relaxation moment of the rehabilitation session and discrete and nonspecific alterations in ventricular repolarization present during all the test’s period and her CF varied from 49 to 93 bpm with an average of 66 bpm.

According to the anthropometric data collected before and after the intervention period, an increase was observed in Grip Strength and Six-minute Walk Test, as we can see in Table 1 below.

**Table 1:** Anthropometric data collected pre and post cardiovascular rehabilitation sessions

	**PRE**	**POST**
Weight (Kg)	62.2	62.3
Height (cm)	158	158
BMI (Kg/m^2^)	24.98	25.02
Waist-to-hip ratio	0.78	0.79
% Fat	31.05	30.11
Flexibility (cm)	225	235
Grip strength (kgf)	0	7.5
Six-minute Walk Test (m)	445	540

In relation to the physiologic responses of CF and BP, which can be observed in Table 2, there were no events. It is important to say that, besides increasing the weight every three sessions, the patient did not reach her HRR in both ergometers in a linear way. It is also necessary to highlight that the patient would arrive in the sessions reporting to be quite sleepy since her daughter would cry a lot during the night, and her sleeping cycle was shorter if compared to the patient’s, according to her own report. Even tough, her responses to BORG never exceed a light level to dyspnea as well as effort perception.

**Table 2:** Maximum and minimum values and the average of physiological responses of CF and BP, facing aerobic exercise, during 21 sessions of cardiovascular rehabilitation

	**Initial**		**EB**				**TM**		**Final**
	**CF i**	**PAS/PAD**	**CF 4’**	**BP 4’**	**CF 10’**	**BP 10’**	**CF 4’**	**CF 10’**	**CF f**
Max	80	110/80	100	120/80	104	110/70	104	116	80
Min	60	80/50	64	80/50	68	80/48	72	72	56
Aver	67.24	90/60	87	95.05/60	88,56	93.7/56.75	86.48	89.13	68

Cardiac frequency (CF) in bpm; Blood pressure (BP) in mmHg; 4’ and 10’ time in minutes and indicates the moments of data taking; EB: exercise bike; TM: treadmill.

## Discussion

The results of this study involving a female patient with SCAD postpartum show that the supervised progressive aerobic exercise was able to cause alterations in the anthopometric values, mainly in the grip strength, flexibility and aerobic capacity.

It was possible to observe the chronicle effects on the CF caused by the aerobic activity, considering that in a normal day with daily activities it changed from an interval of 48 to 110 bpm (average: 70 bpm) to an interval of 49 to 93 bpm (average: 66 bpm). Such fact can be justified by facts like reduction of sympathetic hyperactivity, increase of parasympathetic activity, change in the cardiac bypass or even improvement in systolic function. Together with these benefits, aerobic exercises can improve the endothelial function in the coronary artery of patients with coronary artery disease and documented endothelia disfunction [15-17].

Corroborating these benefits, the patient had an increase of 21% in the walked distance in her 6-Minute Walk Test at the end of 7 weeks of training. Similar data was found in a study with 4,940 men after AMI and/or CABG, subjected to a cardiovascular rehabilitation programme and accompanied for nine years. It can be concluded that the improvement in the walked distance is a strong predictor of coronary artery disease and reflects the assiduity to the treatment [9,18-20].

Moreover, during the cardiac rehabilitation exercises were done in order to maintain and/or get a global stretching, which probably resulted in a change in musculotendinous properties enabling better performance in aerobic capacity and justifying the increase of 4.44% in body flexibility tested in Wells’ Bench.

As an indicator of the body total strength and as a means to evaluate the patient’s progress in a rehabilitation programme, the grip strength can be used, which, in this study, showed expressive improvement, when the patient went from zero to reach 7.5 kgf [11,21]. In addition, the improvement in the upper limbs’ strength can directly affect the individual’s cardiovascular capacity once there is a decrease in the peripheral vascular resistance.

## Conclusion

The report describes the case of a female patient suffering from SCAD postpartum in which cardiovascular rehabilitation brought up positive response in aerobic capacity, flexibility and grip strength, while the anthropometric values were kept in the short-term rehabilitation.

### Consent

The participant signed the term of informed consent to participate in the research, and all the experiment procedures were done with the patient within the ethical standards in Resolution nº 196 from October 10, 1996, from the Brazilian National Health Council. This case study project was submitted to and approved by the Ethical Committee for Researches with Human Beings of FCT/UNESP in Presidente Prudente–Brazil, under the protocol number 79/2011.

## Abbreviations

BP: Blood pressure; SCAD: Spontaneous Coronary Artery Dissection; AMI: Acute Myocardial Infarction; CABG: Coronary Artery Bypass Surgery; DA: Diagonal Artery; CxA: Circumflex Artery; bpm: Beats per minute; BMI: Body Mass Index; CF: Cardiac Frequency; HRR: Heart Rate Reserve.

## Competing interests

The authors declare that they have no competing interests.

## Authors’ contributions

Pinto MC has participated in patient evaluations, data collection, the analysis of data, the conception and design of the paper and been involved in drafting the manuscript. Lorençoni RMR and Camargo RCT have made substantial contributions to the conception and design of the paper and the analysis and interpretation of data, and been involved in drafting the manuscript. Gonçalves ACCR, has made substantial contributions to the conception and design of the paper, and been involved in revising the manuscript. Abreu LC de, Vanderlei LCM, Camargo Filho JCS and Fregonesi CEPT have been involved in revising the manuscript. All the authors have read and approved the final version of the manuscript.
